# Colonization dynamics of a defensive insect ectosymbiont

**DOI:** 10.1098/rsbl.2023.0100

**Published:** 2023-05-10

**Authors:** Ramya Ganesan, Rebekka S. Janke, Martin Kaltenpoth, Laura V. Flórez

**Affiliations:** ^1^ Department of Evolutionary Ecology, Institute of Organismic and Molecular Evolution, Johannes Gutenberg University, 55128 Mainz, Germany; ^2^ Department of Insect Symbiosis, Max Planck Institute for Chemical Ecology, 07745 Jena, Germany; ^3^ Department of Symbiosis, Max Planck Institute for Marine Microbiology, 28359 Bremen, Germany; ^4^ Department of Plant and Environmental Sciences, Section for Organismal Biology, University of Copenhagen, 1871 Frederiksberg C, Denmark

**Keywords:** bacterial colonization, symbiosis, animal–microbe interactions, transmission, motility

## Abstract

Beneficial symbionts are horizontally or vertically transmitted to offspring, relying on host- or microbe-mediated mechanisms for colonization. While multiple studies on symbionts transmitted internally or by feeding highlight host adaptations and dynamics of symbiont colonization, less is known for beneficial microbes colonizing host external surfaces, such as the insect cuticle. Here, we investigate the colonization dynamics of a bacterial symbiont that protects eggs and larvae of *Lagria villosa* beetles against pathogens. After maternal application to the egg surface, symbionts colonize specialized cuticular invaginations on the dorsal surface of larvae. We assessed the colonization time point and investigated the involvement of the host during this process. Symbionts remain on the egg surface before hatching, providing protection. Immediately after hatching, cells from the egg surface colonize the larvae and horizontal acquisition can occur, yet efficiency decreases with increasing larval age. Additionally, passive or host-aided translocation likely supports colonization of the larval symbiotic organs. This may be especially important for the dominant non-motile symbiont strain, while motility of additional strains in the symbiont community might also play a role. Our findings provide insights into the colonization dynamics of cuticle-associated defensive symbionts and suggest alternate or complementary strategies used by different strains for colonization.

## 1. Background

Transmission of microbial symbionts ensures that symbiont-derived benefits such as provision of nutrients, protection against pathogens or breakdown of fastidious polymers or harmful chemicals are sustained through generations [1,2]. Transmission may be horizontal via the environment or unrelated hosts, vertical from parent to offspring or a mixture of both [1]. In insects, some symbionts are localized intracellularly and are vertically transmitted through the germline [3–5]. By contrast, most extracellular symbionts associated with insects experience a phase of environmental exposure during transmission and are acquired after egg hatching, e.g. by probing the symbiont-contaminated egg surface or specialized symbiont-containing secretions [2]. In some cases, the host provides the extracellular symbionts with protection from the environment during transmission [2,6–8]. Other extracellular symbionts in animals are horizontally acquired from the environment each generation [9], and many of these symbionts retain machineries like motility and chemotaxis to enter the host and reach its symbiotic organs [10,11]. Therefore, host and symbiont adaptations can both contribute to successful colonization and establishment [12].

During transmission, the timing of symbiont entry has important implications for the establishment and the evolutionary trajectory of the symbiosis. The timing of colonization can enhance specificity [13] or synchronize host development with the initiation of symbiosis [14]. Ectosymbionts of beewolves [15] and fungus-growing ants [16] are transmitted via maternal provisions or nestmates, respectively, and a colonization window restricts transmission of *Pseudonocardia* symbionts in the fungus-growing ants [16]. However, how precisely the bacteria invade the cuticular structures of adult beewolves and fungus-growing ants has not been described. Similarly, *Lagria villosa* larvae (Coleoptera, Tenebrionidae) carry several strains of *Burkholderia* bacteria in three specialized cuticular invaginations on the dorsal surface of the body [17–20]. However, the process by which symbionts colonize the cuticular structures of the beetles from the egg surface has not been investigated.

As adults, female *L. villosa* beetles host defensive bacterial symbionts in accessory glands associated with the reproductive system [17]. About two million *Burkholderia* cells are smeared onto the egg surface during oviposition, where they produce bioactive secondary metabolites that protect the developing embryo and the larvae from fungal infection [17–19]. Among the different *Burkholderia* strains found in these beetles [17,21], *Burkholderia* Lv-StB, henceforth ‘Lv-StB’, is the most abundant and prevalent strain across individuals, and can produce the antifungal polyketide lagriamide [18]. Yet, Lv-StB remains uncultivated *in vitro* and has a reduced genome in comparison to its close relatives [22]. It is presumably restricted in motility, as functional genes for flagellar biosynthesis are absent from the genome [22]. A closely related strain, *Burkholderia gladioli* Lv-StA, henceforth called ‘Lv-StA’, was isolated from *L. villosa.* It is capable of producing a range of bioactive compounds that protect the insect host and is motile. However, Lv-StA is only sporadically present in field-collected beetles [17–19]. In the congeneric species *Lagria hirta*, it was proposed that the symbionts enter the egg shortly before hatching [23] and colonize the dorsal structures in the embryo as part of the vertical transmission route. However, direct evidence for this route is lacking. Notably, the dorsal structures in *L. villosa* larvae and pupae remain open to the outside [19,20] and the larvae are also capable of acquiring Lv-StA from the environment and successfully transfer them to the adult female glands during metamorphosis [24].

Here, we carried out manipulative assays using the culturable strain, Lv-StA [17], to determine the timing of symbiont entry into the dorsal structures and investigate the efficiency of symbiont acquisition during different time points in early *L. villosa* larvae. To better understand the colonization mechanism of the cuticle-associated symbionts, we additionally tested host involvement in the process by mimicking acquisition with fluorescent beads.

## 2. Methods

### (a) Insect collection and rearing

*Lagria villosa* beetles were collected in soya bean plantations within the state of São Paulo, Brazil (2019, 2022). Adults were fed soya beans, lettuce and kidney bean leaves and kept under a 16/8 h day/night regime at 23–26°C and 55–60% humidity. Water was provided in centrifuge tubes with a cotton plug.

### (b) Preparing *B. gladioli* Lv-StA culture for infection

Lv-StA was grown in King's B (KB) media (soya bean peptone 20 g l^−1^, K_2_HPO_4_ 1.5 g l^−1^, MgSO_4_.7H_2_O 1.5 g l^−1^; agar 15 g l^−1^ for solid media) and incubated at 30°C, 300 rpm for liquid cultures. Bacterial cells were centrifuged at 10 000 rpm for 6 min, and the pellet was washed twice with 1 ml 1× PBS each. The final pellet was resuspended in 500–1000 µl of 1× PBS. The cell concentration was determined using a Neubauer cell counting chamber and adjusted to 2 × 10^6^ cells µl^−1^ in 1× PBS before infecting 2.5 µl of the suspension per egg or larva.

### (c) Estimating the bacterial colonization time point

Lv-StA was inoculated on half of a surface-sterilized, freshly laid egg clutch containing approximately 200–300 eggs (day 0). Egg sterilization was performed as previously described [17]. The other half of the eggs was not infected and used as aposymbiotic control. Six individual mid-time eggs (day 3 after oviposition on day 0), late eggs (day 4), first-instar larvae (day 5) and second-instar larvae (day 6) were collected from three replicate clutches. To quantify the cells that entered the eggs or the larval cuticular structures, individuals were surface washed with 100 µl 1% SDS (thrice) and 1× PBS (twice) to ensure that cells that had not colonized were washed off. Individuals were crushed in 100 µl 1× PBS and diluted (1 : 10 and 1 : 100) before plating on KB agar plates. Lv-StA colony forming units (CFUs) per individual were counted after 24 h.

### (d) Comparing colonization efficiency across time points

Freshly laid eggs from five replicate clutches were surface sterilized and split into four groups. Lv-StA cells were infected on eggs on day 4 (group 1), first-instar larvae on day 5 (group 2), or second-instar larvae on days 6 (group 3) or 7 (group 4). Larvae were collected from the infected groups 24-h post-infection, embedded in 1% agar and stored in 4% formaldehyde for 3–4 months at 4°C. After histological sectioning as previously described [25] we performed fluorescence *in situ* hybridization (FISH).

### (e) Fluorescence *in situ* hybridization

Using FISH [25], we assessed the presence of Lv-StA in the dorsal structures of first and second-instar larvae. Semi-thin sections (8 µm) of larvae were hybridized with fluorescently labelled probes (electronic supplementary material, table S2) and imaged using an AxioImager.Z2 fluorescence microscope (Zeiss, Jena, Germany) or a Leica DMi8 imager (Leica Microsystems, Wetzlar, Germany). We did a single-blinded assessment of microscopy images to compare Lv-StA colonization efficiency before and after hatching. At least three clearly identifiable cells in the dorsal structures were counted as symbiont presence.

### (f) Simulated symbiont transmission from eggs to larvae using fluorescent beads

Individual *L. villosa* eggs were exposed to 2.5 µl (10^6^ beads µl^−1^) of fluorescent microparticles (Sigma Aldrich, latex beads, amine-modified polystyrene, fluorescent red). Given that the size of an Lv-StA cell ranges between 1.0 and 3.0 µm, beads of 1.0 µm mean particle size were used. After hatching, six first-instar larvae were either imaged alive as whole mount (four individuals), after freezing at –20°C, or embedded in 1% agar (two individuals) and fixed in 4% formaldehyde for histological sectioning as described previously [25]. Localization of the beads on eggs and larvae was assessed using epifluorescence microscopy.

## 3. Results and discussion

### (a) When do symbionts enter the larval dorsal structures?

**Figure 1.**
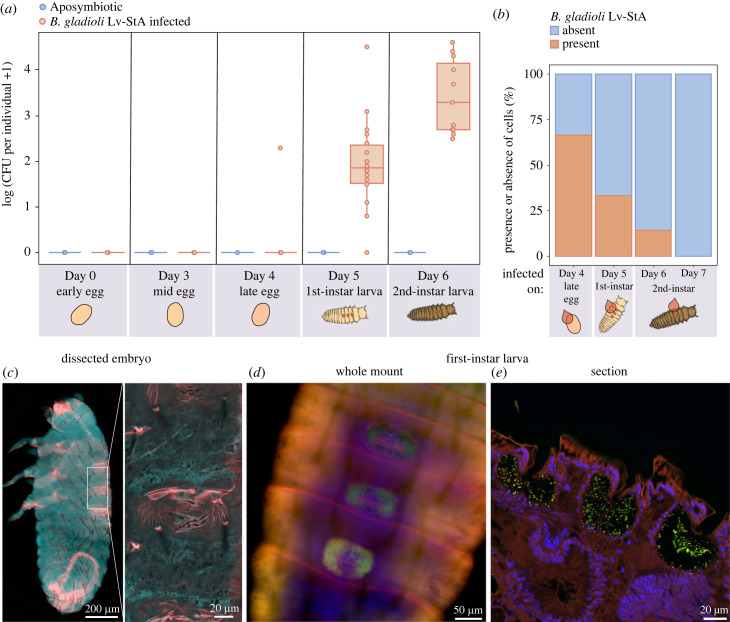
Lv-StA symbionts colonize *Lagria* larvae during or after hatching from the eggs: (*a*) estimation of internal Lv-StA cells per individual in eggs (days 0, 3, 4), first and second-instar larvae. The lack of cells inside eggs shows that the beetles are colonized only in the larval stages during or after hatching (*χ*^2^ = 70.822, d.f. = 4, *p* < 0.001). First instars had significantly fewer cells than second-instar larvae (likelihood ratio = 19.393, *p* < 0.001). (*b*) Percentage of larvae showing presence (orange) or absence (blue) of Lv-StA in the dorsal structures based on FISH images (*χ*^2^ = 7.5208, d.f. = 3, *p* = 0.057). Larvae were collected for FISH 24-h after Lv-StA infection on originally aposymbiotic late eggs (*n* = 6), first-instar (*n* = 6) or second-instar larvae (day 6 (*n* = 7) and day 7 (*n* = 6)). (*c*) FISH of a dissected embryo close before hatching (day 3–4) from a symbiont-infected egg (autofluorescence of the eGFP channel in cyan). The inset shows the larval organs not yet occupied by symbionts. (*d*) Whole-mount FISH image of a field-collected first-instar larva showing Lv-StB within the organs. (*e*) Lv-StA cells in the three dorsal organs of an infected first-instar larva. (*d*,*e*) *Burkholderia*-specific staining is shown in green, eubacteria in red, host cell nuclei in blue.

Female *L. villosa* adults vertically transmit on average 2 × 10^6^ symbiont cells onto the egg surface [17], where they remain over a period of 4 days until hatching. In larvae, the symbionts are housed in three invaginations of the dorsal cuticle [17–20]. After transmission onto the egg surface, it is possible that (a) symbionts enter the late egg to colonize the dorsal structures of the embryo as described for the related species *L. hirta* [23], or (b) larvae acquire the symbionts from the egg surface during or after hatching. To evaluate which route Lv-StA follows in *L. villosa*, we infected freshly laid eggs with 2 × 10^6^ cells and assessed the presence of cells internalized in eggs or larvae by CFU counting after removal of residual symbionts on the surface. We did not detect any colonies on the plate when we sampled eggs, with a single exception that may have resulted from incomplete surface sterilization. However, significantly higher CFU counts were obtained from first-instar larvae (*n* = 18) after hatching (*χ*^2^ = 70.822, d.f. = 4, *p* < 0.001), and, on average 2044 cells (min = 0, max = 33 600) (figure 1*a*, electronic supplementary material, methods) were estimated to colonize the dorsal structures. In second-instar larvae (*n* = 11), CFU counts amounted to 9431 on average (min = 290, max = 40 300), which is significantly higher than in first-instar larvae (likelihood ratio = 19.393, *p* < 0.001) (figure 1*a*, electronic supplementary material, methods). This indicates that symbionts colonize during or after hatching, colonizing cell number is highly variable across different host individuals and symbiont titer increases over time. Complementary FISH experiments on a dissected embryo and early first-instar larvae support the finding that bacteria are absent in the embryo and colonize the structures after hatching (figure 1, electronic supplementary material, supp.figure S1*a*,*b*). In conclusion, colonization occurs post-hatching for Lv-StA in *L. villosa* and differs from Stammer's observation of symbionts in *L. hirta* [23], however, we cannot exclude the possibility that other strains associated with *L. villosa* colonize at an earlier time point.

Previous observations indicate that most symbiont cells remain in the dorsal structures during larval moults, although some can be released from the structures and adhere to the moulted exuvia [19]. Since laboratory-reared second-instar larvae often consume moulted exuviae, we evaluated a potential overestimation of colonizing cell numbers per individual. Indeed, FISH images from eight out of 10 sampled second-instars show the cells adhering to the exuvia within the gut (electronic supplementary material, supp.figure S1*c*,*d*). Therefore, CFU counts in infected second-instars possibly represent the sum of cells in the dorsal structures and those present in the gut. However, the cells in the gut are probably transient as they are not consistently present at later time points and do not seem to adhere to the gut lining.

### (b) Symbiont acquisition efficiency declines over time

Aposymbiotic larvae are capable of acquiring Lv-StA symbionts when repeatedly exposed to infected leaf litter [24]. To understand the efficiency of symbiont acquisition in younger larvae in a defined time frame after hatching, we performed inoculations on late eggs (day 4), first-instar (day 5) and second-instar larvae (days 6 and 7) and assessed the presence or absence of Lv-StA 24-h post-exposure. A comparatively low number of samples could be assessed through the laborious microscopy technique, which limits the statistical power of this dataset. However, there is a clear and marginally significant trend for lower efficiency of symbiont acquisition with increasing age of the larvae (*χ*^2^ = 7.5208, d.f. = 3, *p*-val = 0.057) (figure 1*b*, electronic supplementary material, methods). This suggests that symbiont acquisition from the egg surface is more likely to succeed compared to acquisition by larvae one or more days after hatching. While this early time window might impose constraints, previous studies suggest that horizontal acquisition is also possible later in larval development [24]. An environment with high Lv-StA abundance and increased frequency of exposure may encourage horizontal acquisition in larvae [24]. However, in natural populations, priority effects and inter-microbial interactions may also hinder entry by a successive colonizer [26–29].

### (c) Are host movements enough for symbiont acquisition?

**Figure 2.**
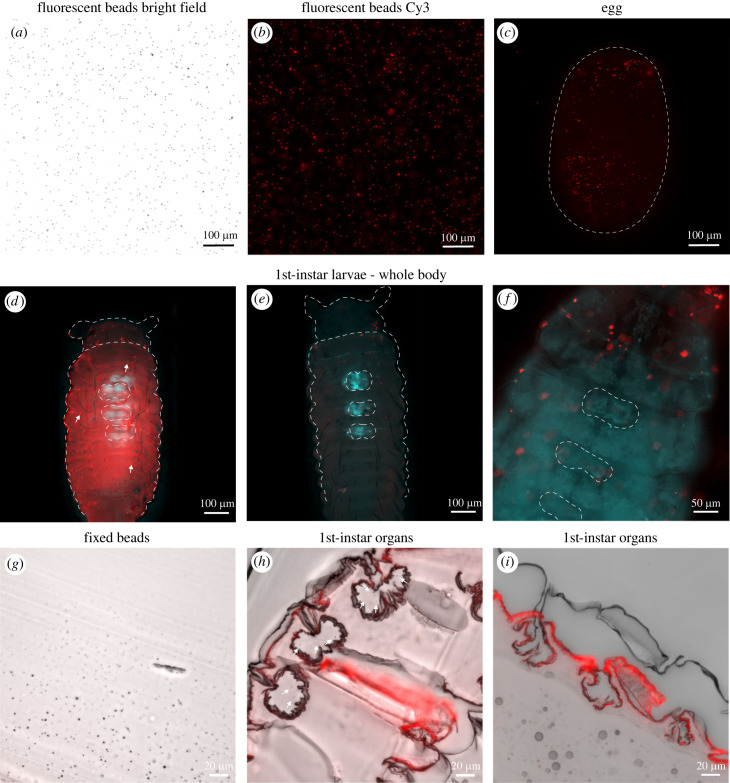
Simulating transfer of symbionts from eggs to the larval surface using fluorescent beads: (*a*,*b*) fluorescent beads in a PBS suspension imaged with bright-field microscopy(*a*) and in the Cy3-channel (*b*). (*c*) A beetle egg after inoculation with fluorescent beads. (*d*,*f*) Three whole first-instar larvae that hatched from eggs carrying beads on the outer surface. The individual in (*d*) shows autofluorescence in the Cy3 channel, most likely due to early cuticle melanization. White arrows aid in distinguishing the beads. (*c*–*f*) Dotted lines indicate specimen profiles and the dorsal structures based on the DAPI signal (not shown) or EGFP channel (cyan) autofluorescence of the larval tissues. (*g*) A section of a 1% agar block with fixed beads. The beads lost fluorescence when fixed in 4% formaldehyde and dehydrated but are visible with bright-field microscopy. (*h*,*i*) Sagittal section of a hatched first-instar larva after bead inoculation showing a few (arrowheads in (*h*)) or no beads (*i*) within the dorsal structures. Overlay of bright-field microscopy (black and white) and Cy3-channel (red). The red signal corresponds to cuticle autofluorescence.

The dominant symbiotic strain, Lv-StB is likely incapable of swimming motility, as it lacks genes for flagellar synthesis and chemotaxis [22]. It is puzzling how a strain lacking a flagellum can successfully colonize and dominate the host-associated community, given that entry into the dorsal structures of the host is necessary [19,20]. By simulating symbiont transmission using fluorescent beads (figure 2*a*,*b*), we tested if the larva's movements while hatching (electronic supplementary material, video S1) are sufficient to direct particles, and thus potentially cells of comparable size, into the dorsal structures. Beads suspended in 1× PBS at a concentration comparable to the natural cell number found on *L. villosa* eggs [17] were applied to the egg surface, where we confirmed their presence (figure 2*c*). After hatching, the surface of first-instar larvae was covered with the beads, including regions close to the symbiotic organs (figure 2*d*–*f*). While we could not detect a large number of beads within the dorsal structures in whole larvae (figure 2*d*–*f*), very few beads were occasionally seen in histological sections (figure 2*g*–*i*). This suggests that immotile beads can successfully translocate from eggs onto the larval outer surface, and some passively reach the symbiotic structures.

In a complementary experiment to visualize the colonization process, eggs were infected with GFP-tagged motile Lv-StA (electronic supplementary material, methods, electronic supplementary material, table S1), and hatching larvae were imaged under a light-sheet microscope (electronic supplementary material, methods). Although we could not yet observe cells entering from the egg surface into the dorsal structures, we visualized motile Lv-StA in the dorsal structures of the first-instar larva shortly after hatching (electronic supplementary material, video S2, electronic supplementary material, methods).

Since only a few immotile beads seem to enter the organs, and the light sheet imaging (electronic supplementary material, video S2, electronic supplementary material, methods) shows actively moving Lv-StA cells in the larval organs, it is probable that some symbiont-mediated cellular mechanisms, likely including motility, are important factors enhancing colonization efficiency. It is intriguing how an immotile Lv-StB colonizes and dominates the symbiotic organs after the larvae hatch. We suspect that Lv-StB might make use of an alternative approach, like inter-bacterial hitchhiking [30] or a host-controlled mechanism to navigate into the organs. Alternatively, a few immotile cells of Lv-StB entering the organs by chance due to larval movements may have a competitive advantage over Lv-StA in utilizing host-provided resources, leading to strain dominance. These results indicate that microbial entry into the structures may initially be non-specific and it is probable that host selection or inter-bacterial competition determine the specificity of the association.

## 4. Conclusion

Symbiont colonization of a host usually involves considerable changes in effective population size, translocation to a new habitat and re-establishment of the host–microbe interaction. As such, it is a key stage determining symbiosis stability and evolutionary fate. We show that *L. villosa* larvae can acquire cells from the surface of the egg, most likely when the larva brushes against the egg surface as it hatches. Infection experiments at different time points indicate that direct transfer of symbionts from the egg surface is a more effective colonization strategy than horizontal acquisition during later larval instars. However, further experiments investigating the mechanics of colonization with the most dominant strain, Lv-StB, and interactions between multiple symbiont strains during colonization are necessary to understand the natural strain dynamics. Our results also reveal that host movements are sufficient for symbionts to spread over the larval external surface, but navigation into the specialized dorsal structures is probably aided by symbiont molecular factors that are yet to be investigated.

## Data Availability

The data are provided in the electronic supplementary material [31].
